# Vulnerable Populations Perceive Their Health as at Risk from Climate Change

**DOI:** 10.3390/ijerph121214994

**Published:** 2015-12-05

**Authors:** Karen L. Akerlof, Paul L. Delamater, Caroline R. Boules, Crystal R. Upperman, Clifford S. Mitchell

**Affiliations:** 1 Center for Climate Change Communication, George Mason University, Fairfax, VA 22030, USA; cboules@masonlive.gmu.edu; 2 Department of Geography and GeoInformation Science, George Mason University, Fairfax, VA 22030, USA; pdelamat@gmu.edu; 3 University of Maryland School of Public Health, College Park, MD 20742, USA; cromeo@umd.edu; 4 Maryland Department of Health & Mental Hygiene, Baltimore, MD 21201, USA; cliff.mitchell@maryland.gov

**Keywords:** vulnerable populations, health risk perceptions, climate change communication

## Abstract

Climate change is already taking a toll on human health, a toll that is likely to increase in coming decades. The relationship between risk perceptions and vulnerability to climate change’s health threats has received little attention, even though an understanding of the dynamics of adaptation among particularly susceptible populations is becoming increasingly important. We demonstrate that some people whose health will suffer the greatest harms from climate change—due to social vulnerability, health susceptibility, and exposure to hazards—already feel they are at risk. In a 2013 survey we measured Maryland residents’ climate beliefs, health risk perceptions, and household social vulnerability characteristics, including medical conditions (*n* = 2126). We paired survey responses with secondary data sources for residence in a floodplain and/or urban heat island to predict perceptions of personal and household climate health risk. General health risk perceptions, political ideology, and climate beliefs are the strongest predictors. Yet, people in households with the following characteristics also see themselves at higher risk: members with one or more medical conditions or disabilities; low income; racial/ethnic minorities; and residence in a floodplain. In light of these results, climate health communication among vulnerable populations should emphasize protective actions instead of risk messages.

## 1. Introduction

Public perceptions of climate change risk have primarily been explained by political and cultural worldviews, awareness of physical changes in the environment, and understanding of the scientific evidence [1,2]; the role of vulnerability in shaping people’s assessment of the threat has been less studied [3]. Indeed, whether vulnerability specifically due to health status influences individuals’ perceptions of their climate change risks has been little explored [2], but is particularly of relevance for climate adaptation planning. Public health organizations have shown an increasing interest in communicating with vulnerable populations in order to promote successful adaptation strategies for these health threats [4]. Indeed, the Centers for Disease Control and Prevention (CDC) established a Climate and Health Program in 2009 [5]. Maryland participates in the CDC Climate-Ready States and Cities Initiative in which it is tasked with assessing state health vulnerabilities and potential interventions, developing and implementing a climate and health plan, and conducting ongoing activity evaluation.

Climate change both directly and indirectly affects human health [6,7]. Direct impacts result in human injuries; illnesses and deaths related to extreme weather events and changes in weather patterns; rises in infectious diseases due to changes in vector-pathogen relations; and increased disease burdens from declines in water and air quality. Indirect impacts include impaired food security and nutrition as a result of changes in crop yields, as well as displacement and loss of livelihoods leading to negative health effects.

Climate change vulnerability represents the propensity to be adversely affected under these conditions of risk and can be deconstructed into three components: sensitivity, exposure, and the capacity to adapt [7]. Populations vary in degree of vulnerability depending on these factors. Those with higher sensitivity include women, children, the elderly, and those with chronic illnesses and disability. Those in urban heat islands, floodplains, and coastal communities are at greater exposure, and socioeconomically disadvantaged groups may have both greater exposure and less capacity for adaptation [6,8,9,10].

In 2014, a majority of Marylanders recognized that people with medical conditions (59%) and the elderly (55%) are very vulnerable to the potential health impacts of climate change; they were less likely to identify young children and people with low incomes as very vulnerable (42% and 36%, respectively) [11]. Moreover, Marylanders were more likely to say that people in the United States generally are “very vulnerable” to the health effects of climate change (31%) or even people in Maryland (21%), than they themselves (11%) or those in their households (13%).

Public awareness and knowledge of climate health risks are an important component of climate adaptation [12,13]. Nevertheless, there has been little research on people’s perceptions of health risks from climate change either in the United States or abroad [14,15,16,17,18]. Surveys from the United States, Canada and Malta that assessed public understanding of the links between climate change and human health have found that less than half of American respondents believe that they themselves are at risk from climate impacts [18,19]; conversely, rates of concerned respondents are higher in Canada and Malta [19]. The frequency of cited health impacts is greater for multiple choice responses or specific prompts than open-ended questions [14,18,19]. This suggests that although people believe climate change will cause declines in public health, it is not a highly salient issue.

The feeling of being at risk has long been known to be influenced by social vulnerability, especially gender and race [20,21]. Previous research has operationalized social vulnerability as a combination of perceived social and economic discrimination, poor health, and reduced access to medical care [22]. Most studies on the relationship between vulnerability and climate change risk perceptions have focused on the physical environment instead: direct experience of changes in weather and climate, and exposure to hazards based on location and current or projected conditions [3,23]. Moreover, the results at times have been conflicting. Personal experiences of flood events and higher temperatures have been correlated with higher risk perceptions of climate change [24,25], though not consistently [26,27].

To our knowledge, the influence of health susceptibility on personal and household perceptions of climate change health risk has not been studied, particularly in combination with two of the most common climate impacts that vulnerable communities in the United States are likely to face: extreme heat in urban areas, and flooding [10]. In this study we hypothesize, based on previous literature, that general health risk perceptions, political ideology, and climate beliefs will strongly predict perceptions of climate change as a personal and household health threat. Furthermore, we pose two research questions:

RQ_1_: Do measures of social vulnerability and health susceptibility explain unique variance in perceived vulnerability to the health effects of climate change above and beyond other known covariates?
RQ_2_: Do measures of exposure to risk of flooding and urban heat explain unique variance in perceived vulnerability to the health effects of climate change above and beyond other known covariates?

To investigate the relationship between perceptions of personal and household climate health risk and vulnerability due to social status, health, and location, we employ data from a mail survey of more than 2000 Maryland residents. These results are paired with secondary data sources for 100-year floodplain designations and urban *versus* rural locations as a proxy for higher temperatures due to heat island effects.

## 2. Methods

### 2.1. Data Collection and Treatment

Data for this study were collected as part of a survey fielded from 28 March to 4 June 2013 with 6401 households in Maryland. The questionnaire was sent to residents by the Maryland Department of Health and Mental Hygiene. The instrument included measures of health and environmental risk perceptions, preferences for energy sources, pro-environmental behaviors, state climate and energy policy preferences, and climate change beliefs. The survey consisted of 55 questions and took approximately 20 min to complete. Using stratified sampling, households were randomly selected from a Survey Sampling International address database of residents for each of four regions of the state (Table S1). The survey was fielded from 28 March to 4 June 2013. Each household was sent up to four mailings: An announcement letter introducing the survey (March 28), a copy of the survey with a $2 bill thank you (April 1), a reminder postcard (April 13), and a follow-up survey (April 29). In order to achieve randomization of respondents within each household, the person with the most recent birthday was asked to complete the survey. The methodology is based on a commonly used mail survey technique developed by Dillman and colleagues [28]. The study was reviewed by Institutional Review Boards for both George Mason University and Maryland Department of Health and Mental Hygiene.

In weighting the dataset for public crosstabular reporting, 146 respondents were dropped due to lack of regional, gender, education or age identification (6.4% of the initial sample of 2272). The final sample of respondents was 2126 with a response rate of 38%. This response rate compares favorably to other similar university studies using the same methodology [29]. While survey response is declining across all methodologies with below 10% response rates normative for online panels and telephone samples utilized by researchers [30,31,32], mail surveys have seen less of a drop [33]. Mail survey response rates can achieve between 40%–70% response rates when incentives of $5 or more are included and the survey sponsor is well-known to the respondents [33].

To accommodate the regional sampling design and the causal nature of the study, the data were weighted solely to readjust for state population density distributions [34]. Missing data ranged between 1%–9.5% for each of the independent variables in the analysis (Table S2), and between 1.2%–2.6% for the dependent variable. All missing survey data, with the exception of race and ethnicity, were substituted using hot deck imputation, a strategy for handling missing data that is widely employed by government statistics agencies, survey organizations, and academic researchers [35]. In hot deck imputation, respondents matched on a set of variables to the case with the missing value are randomly sorted; the missing value is replaced with that of the nearest neighbor [36]. This single imputation technique is distinct from multiple imputation [37]. The matched variables were gender, age and education, for which there was no missing data but expected differences on attitudinal measures and income. For more information on hot deck imputation with application to public health see Hawthorne and Elliott [38].

Sample bias can be assessed by comparing sample characteristics to those of the population, testing for differences between early and late waves of responders and extrapolation of linear trends over time, and contrasting the characteristics of respondents to non-respondents on variables of research relevance [39]. We performed two tests for bias. We compared the sample to known Census distributions for the state and Gallup reports of political ideology frequencies, and split 20% of the sample into early and late responders on the primary variable of interest—perceived vulnerability to climate change health risk—to test for differences between the groups. We do not have data on non-respondents, but late survey responders can be assumed to resemble non-respondents and function as a proxy [39]. Testing for mean differences between the first 10% of responders (*n* = 212) and last 10% (*n* = 212), we find no statistically significant difference in the dependent variable (*M*_first10%_ = −0.12; *M*_last10%_ = 0.01; *t*(422) = −1.40, *p* = 0.16). Compared to U.S. Census distributions for Maryland, the final sample is slightly older, more female, and better educated (Table 1). The differences in sample characteristics from the known population values suggest at possible bias in the regression estimates, but the finding that there appears to be insignificant differences between respondents and a proxy for non-respondents on the primary variable of interest implies that these are likely to be limited.

Addresses for respondents were geocoded, then overlain with urban/rural and 100-year floodplain maps in a Geographic Information System (GIS).

**Table 1:** Demographics of survey respondents compared with Maryland population.

		Survey Respondents	*n*	U.S. Census
				Gallup *
Female		62.3%	2126	51.5%
65 years +		25.1%	2126	13.4%
Education	Less than high school	1.8%	2126	11.3%
	High school or equivalency test	26.1%		45.6%
	2-year associate’s degree or trade school	16.7%		6.3%
	4-year college degree	24.7%		20.1%
	Advanced degree beyond 4-year degree	30.7%		16.7%
Income	Median household	$70,000–$89,999	1986	$73,538
African American or black		21.6%	2068	30.1%
Hispanic or Latino		3.5%	2023	9.0%
Political ideology	Conservative	30.8%	2073	32% *
	Moderate	37.5%		38% *
	Liberal	31.7%		25% *

Frequencies represent regional samples weighted to statewide population distributions; *n* sizes are unweighted totals. Gallup includes a no-response category which accounts for an additional 5% of the sample [40]; * denotes the data in the table that is from that source.

### 2.2. Dependent Measures

The primary dependent variable—perceived personal and household health vulnerability to the effects of climate change—represents factor scores derived from three individual measures (α = 0.78) (Table 2). Respondents answering “don’t know” to their level of vulnerability on one or more of the three risk measures (19%) were dropped in the calculation of the measure due to the non-continuous nature of the data. The exploratory factor analysis extracted one component which explains 54% of the variance across the three variables. The communalities are 0.40, 0.69, and 0.53 respectively. The Kaiser-Meyer-Olkin measure of sampling adequacy is 0.68, above the recommended threshold of 0.6, and Bartlett’s Test of Sphericity, testing the equivalence of the variances, is statistically significant (*p* < 0.001).

In a second analysis, predictors of “don’t know” responses were evaluated using just one of the questions from the composite measure: “How vulnerable—if at all—are the people living in your immediate household, including yourself, to potential health impacts of climate change?”

### 2.3. Covariates

*Political ideology.* Respondents were asked “Generally speaking, do you think of yourself as politically … .” Responses ranged from very conservative (1) to very liberal (5) (Table S2).

*Perceived general health risk.* In order to account for overall differences in risk perception levels, we utilized regression scores from a six-item factor analysis of respondents’ perceived risk from general threats to their health and wellbeing, including second-hand smoke, air pollution, obesity, flu epidemics, chemicals, and polluted water (*α* = 0.82) (Table S3). In the exploratory factor analysis, one component explained 45% of the variance across the six variables. The communalities—percent variance of the variables explained by the extraction—are 0.42, 0.51, 0.30, 0.51, 0.58, and 0.40 respectively. The Kaiser-Meyer-Olkin measure of sampling adequacy is 0.84, above the recommended threshold of 0.6, and Bartlett’s Test of Sphericity, testing the equivalence of the variances, is statistically significant (*p* < 0.001).

“Don’t know” responses were coded as missing; they represent between 1.1% and 2.4% of responses for each measure and 5.8% total across all 6 measures. “Don’t know” values are left missing and not imputed. Values for other missing data were imputed. They account for between 1.3 to 1.8 % of each response for a total of 7.1% across all six measures.

**Table 2:** Perceived vulnerability to climate change health risks (Dependent Variable): Factor score variables.

	*Frequencies*	*n*	*M*	*SD*
Below is a list of potential risks to people’s health. How much of a risk do you feel each currently poses to your own health? (Climate change) *Don’t know, 2.7%*	No risk at all (1), 14.8%	2066	2.61	0.99
	Minor risk (2), 31.5%			
	Moderate risk (3), 31.8%			
	Major risk (4), 21.9%			
How much do you think climate change will harm … (you personally)? *Don’t know, 8.5%*	Not at all (1), 17.6%	1944	2.54	0.96
	Only a little (2), 26.7%			
	A moderate amount (3), 39.7%			
	A great deal (4), 16.0%			
How vulnerable—if at all—are the people living in your immediate household, including yourself, to potential health impacts of climate change? *Don’t know, 8.7%*	Not at all vulnerable/No potential climate change health impacts (1,5), 17.4%	1941	2.51	0.94
	Only a little vulnerable (2), 29.0%			
	Moderately vulnerable (3), 39.3%			
	Very vulnerable (4), 14.4%			

The regional sample data has been reweighted to state population distributions; n sizes are unweighted totals.

### 2.4. Independent Measures

*Climate change beliefs.* Climate change certainty is measured with two questions: “Do you think that climate change is happening?” (yes, no, don’t know); and “If you answered either yes or no, how sure are you?” (not at all sure, somewhat sure, very sure, extremely sure). The two measures are combined into a 9-point scale ranging from (1) extremely sure climate change is not happening to (9) extremely sure climate change is happening. A scale comprised of four dichotomous items represents the number of perceived local climate health impacts that respondents believe will occur in Maryland. The question “Which—if any—of the following health problems will become more common in Maryland in the future because of climate change?” includes four response categories: respiratory and breathing problems; infectious diseases such as West Nile virus; heat stroke; and injuries from storms or other extreme weather events. The responses were added to create an additive scale from 0–4 correct responses.

*Social vulnerability and health susceptibility.* Seven measures represent socioeconomic characteristics that capture social vulnerability and health susceptibility: gender, income, education, racial and/or ethnic minority group, age 65 years or older, and household members with chronic medical conditions and/or disabilities (Table S2). Household pre-existing medical conditions and disabilities were measured thus: “Has a doctor ever diagnosed you or another member of your household with the following conditions?” Survey participants were given a list of five conditions: coronary heart disease; obesity; diabetes; respiratory illness, including asthma; or a physical or mental disability. Responses were summed into a composite score from 0 to 5, then split into a three-category variable: (0) no diagnosed medical conditions; (1) one diagnosed condition; (2) two or more diagnosed conditions. Age is coded as a dichotomous variable for those age 65 or greater.

*Exposure to hazards.* Flood risk is captured by household location within a 100-year floodplain using 2015 FEMA Digital Flood Insurance Rate Map data obtained from the state of Maryland [41]. Exposure to higher temperatures due to location in a highly populated urban area [5] is represented by the percent of the population designated urban in U.S. Census-designated zip code tabulation areas in 2010 [42]. The Census classifies urban areas primarily based on population density. While recent studies may use modeled climate data estimates for measurements of urban heat island intensity [43], in this study we employ the Census urban-rural designation as a rough proxy for exposure to higher temperatures due to the urban heat island effect based on early research demonstrating a direct relationship between population density and temperatures [44].

### 2.5. Statistical Analyses

The analysis consists of two steps. First, we used hierarchical multiple regression (OLS) with blockwise entry [45] to model perceptions of personal and household climate change health risk using the following sets of independent variables as predictors: climate beliefs, social vulnerability and health susceptibility, and exposure to risk from climate change impacts. Political ideology and a composite measure of general health risk perceptions are covariates.

Second, we performed a logistic regression with the same control and predictor variables as in the hierarchical multiple regression in order to assess which factors best predict those 8.7% of respondents who say they do not know what their and their household’s health vulnerability is to climate change. Low understanding of climate risks, and corresponding risk perception, have been assumed among the most vulnerable populations of the United States. For example, “do not know” is a characteristic response of one of the Global Warming’s Six Americas audiences typified by low income and education levels, the “Disengaged,” to questions about the effects of climate change [46].

All analyses, unless otherwise indicated, were run using SPSS (v. 21). All GIS and mapping analyses were performed using ArcGIS (v. 10.2).

## 3. Results

**Figure 1:**
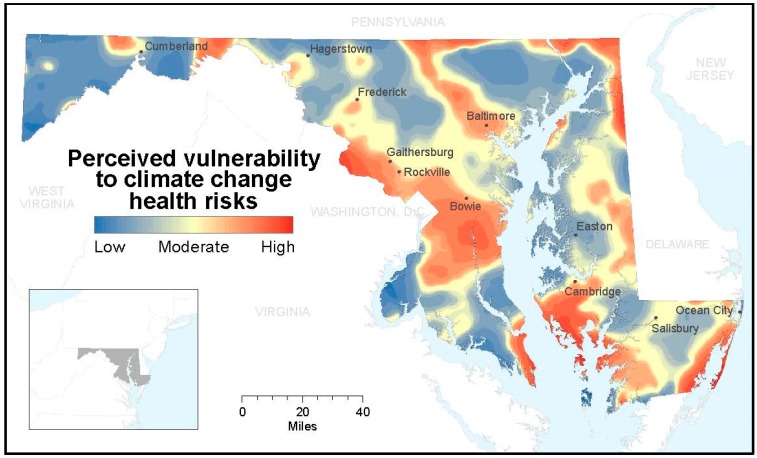
Geographic distribution of perceived vulnerability to climate change health risks demonstrated as a relative distribution by interpolating the factor score variable.

In 2013, more than half of Marylanders (53%) said that they and their households are moderately or very vulnerable to the potential health effects of climate change (Table 2). Only 17% of residents said that there would be no health impacts from climate change, or that they and members of their household were not vulnerable. There is substantial variation in climate health risk perceptions across the state, with mapping of the aggregate dependent climate health risk variable revealing specific regions of higher and lower risk perception than others, such as some coastal and urban areas (Figure 1). Visual interpretation of the mapped results suggests that some of the higher areas of perceived risk are both more exposed to physical risks and have greater social vulnerability. The low-lying regions of the DelMarVa peninsula, the geographic region located between the Chesapeake Bay and Delaware Bay, are prone to flooding and at risk from projected inundation from sea-level rise, especially south of Cambridge [47]. Urban heat islands cluster around the cities of Washington, D.C. and Baltimore [48]. Areas of the state with medium to high social vulnerability include almost all of Maryland’s Eastern Shore on the DelMarVa peninsula; Baltimore City and the surrounding county; Prince George’s County to the immediate east and north of Washington, D.C. (see Bowie on the map); the southernmost county on the eastern side of the Chesapeake Bay, St. Mary’s; and the state’s panhandle north of West Virginia [49]. Further research is needed to empirically test these perceived spatial correlations.

### 3.1. Regression Model Results Predicting Perceived Health Vulnerability

As hypothesized, the covariates—general health risk perceptions and political ideology—account for a sizeable percentage of the variance in perceptions of vulnerability to climate health harms, 25% (adj. R^2^). Also as hypothesized, climate beliefs—certainty that climate change is occurring and an understanding of specific climate change health risks in Maryland—also greatly contribute to the explanatory capacity of the model, almost doubling it to 48%. The additional explanatory power of variables representing social vulnerability and hazard exposure is comparatively low, yielding an additional 3 percentage points in adjusted R^2^.

Our research questions posed whether social vulnerability and health susceptibility (RQ_1_), and risk exposure (RQ_2_), influence perceptions of climate change’s health threat. The results suggest these effects exist. Addressing our first research question, five of the seven factors for social vulnerability and health susceptibility are significant predictors of climate change health risk perceptions in a direction that would suggest that people who are more vulnerable feel themselves to be at higher risk, even holding constant general health risk perceptions, political ideology, and climate change beliefs (Table 3). Having one or more chronic medical conditions, being a member of a community of color (African American/black, Hispanic/Latino), and having less income are significant predictors of higher risk perceptions; being female is of near significance (*p* = 0.05). Higher education levels, while not associated with increased vulnerability to climate risks, also predict higher health risk perceptions. Being elderly is not a significant predictor.

The results for exposure—the second research question—are split. In the final model, flooding is a significant predictor, while the proxy for urban heat is not. The social vulnerability and exposure variables were added to the regression in the final two regression models to demonstrate that they account for unique variance above and beyond general risk perceptions, climate beliefs, and political ideology. If the order of the regression models are reversed, with social vulnerability and exposure entered first, they explain 14% of the variance in the dependent variable as measured by adjusted R^2^.

**Table 3:** Predictors of perceived vulnerability to climate change health risks.

			Model 1	Model 2	Model 3
			β (95% CI)	β (95% CI)	β (95% CI)
Covariates	Political ideology	**0.25** *** (0.20, 0.29)	**0.05** ** (0.01, 0.09)	**0.05** * (0.01, 0.08)	**0.04** * (0.01, 0.08)
	Non-climate health risk perceptions	**0.41** *** (0.37, 0.45)	**0.30** *** (0.26, 0.33)	**0.28** *** (0.24, 0.31)	**0.28** *** (0.24, 0.31)
Climate beliefs	Climate change belief certainty		**0.32** *** (0.28, 0.36)	**0.30** *** (0.27, 0.34)	**0.30** *** (0.26, 0.34)
	Perceived climate health impacts in Maryland		**0.31** *** (0.27, 0.35)	**0.28** *** (0.24, 0.32)	**0.28** *** (0.24, 0.32)
Social vulnerability & health susceptibility	Female			**0.03** * (0, 0.07)	**0.03** ^#^ (0, 0.07)
	African American/black			**0.12** *** (0.09, 0.16)	**0.12** *** (0.09, 0.16)
	Hispanic/Latino			**0.04** * (0.01, 0.07)	**0.04** * (0.01, 0.07)
	Income			**−0.06** ** (−0.10, −0.02)	**−0.06** ** (−0.10, −0.02)
	Education			**0.04** * (0.01, 0.08)	**0.04** * (0, 0.08)
	Elderly (Age 65+)			−0.02 (−0.05, 0.02)	−0.02 (−0.05, 0.02)
	Chronic disease/disability			**0.05** * (0.01, 0.08)	**0.04** * (0.01, 0.08)
Exposure	100-year floodplain				**0.05** ** (0.02, 0.08)
	Urban heat proxy				0.03 (−0.01, 0.06)
	Adjusted R^2^	0.25	0.48	0.50	0.51
	∆ F	**281.79** ***	**387.06** ***	**13.18** ***	**5.23** **

*n* = 1727; boldface standardized coefficient indicates significance (*p* < 0.05 *; *p* < 0.01 **; *p* < 0.001 ***); *p* = 0.05 ^#^.

### 3.2. Logistic Regression Model Results Predicting Those Unaware of Climate Health Risks

We also find that some—but not all—of the factors for social vulnerability and health susceptibility influence whether or not people say they know their climate change health risks (Table 4). Of the more than 2000 survey respondents, 183 said that they did not know whether they and their household were vulnerable to the health effects of climate change. Those who are at higher health risk from climate change—such as those with medical conditions, women, and people from communities of color—are more likely to say they know whether they are at risk. A number of characteristics not associated with greater risk—knowledge (level of education, and information about state climate health impacts) and income—also decrease the likelihood that someone will say they do not know whether they and their household are at risk from the health effects of climate change.

The predictors in the logistic regression model all have odds ratios of less than 1, signifying that as each of these variables increases, individuals are less likely to report “don’t know” to personal and household climate change health vulnerability (Table 4). As each measure increases by one, holding all other variables constant, the change in the odds that the respondent will say they do not know, *versus* they say they know, is 0.55 for females, 0.81 for income, 0.69 for education, 0.62 for African Americans, 0.33 for Hispanics and Latinos, 0.72 for medical conditions, and 0.81 for Maryland climate health impact perceptions. Another way of saying this is that these groups are each less likely than their comparison groups to say that they do not know whether they are vulnerable. Women are 81% less likely than men; those with higher income levels are successively 23% less likely; those with higher education levels are successively 45% less likely; African Americans are 61% less likely compared to other races; Hispanics and Latinos are 199% less likely compared to those not of those ethnicities; those with higher levels of medical conditions and/or disabilities are successively 39% less likely; and those with higher perceptions of Maryland climate health impact are successively 24% less likely. The results should be interpreted with caution, however, as logistic regression has well-known problems in modeling rare events [50].

**Table 4:** Predictors of “don’t know” responses to perceived climate change health vulnerability.

	*b*	*SE*	*p*	*Odds Ratio* (95% CI)
*Constant*	**2.04**	0.46	<0.001	
Perceived climate health impacts	**−0.21**	0.06	<0.001	0.81 (0.72, 0.91)
Female	**−0.60**	0.19	<0.01	0.55 (0.38, 0.79)
African American/black	**−0.47**	0.18	<0.01	0.62 (0.44, 0.88)
Hispanic/Latino	**−1.10**	0.34	<0.01	0.33 (0.17, 0.65)
Income	**−0.21**	0.04	<0.001	0.81 (0.74, 0.88)
Education	**−0.37**	0.08	<0.001	0.69 (0.59, 0.80)
Chronic disease	**−0.33**	0.11	<0.01	0.72 (0.58, 0.89)

Model χ2 (7) = 149.900, *p* < 0.001; pseudo R^2^ values, Cox and Snell, 0.068; Nagelkerke, 0.153. “Don’t know” *n* = 183; “other” *n* = 1939. The boldface unstandardized coefficients indicate statistical significance. The final logistic regression model represents only those predictors with coefficients significant at *p* < 0.10 in the original model, entered hierarchically in blocks.

## 4. Conclusions

This study finds that some populations at higher risk for health impacts associated with climate change, and greater exposure, already see themselves as more vulnerable than members of the public at large. Having one or more chronic medical conditions, being a member of a community of color (African American/black, Hispanic/Latino), location in a floodplain, and having less income are significant predictors of higher risk perceptions; being female is of near significance. This result aligns with previous research that has found that vulnerable members of society feel at greater risk to environmental threats [20,21]. Indeed, those most at risk from climate change are generally those who are susceptible to any number of environmental and societal harms. Yet the finding is surprising for two reasons: (1) the effects of climate change are arguably more abstract and long-term compared to the more visible and immediate environmental hazards, such as natural disasters, chemical manufacturing, and nuclear power, which have been the focus of other studies; and (2) the effect of being vulnerable to climate health impacts remained identifiable, even when general health risk perceptions were held constant.

A cursory interpretation of current audiences developed to explain climate change beliefs, risk perceptions, and policy support would suggest that people who are most socially vulnerable are those who are also most unsure about the effects of climate change [46]. This study shows the need for a more nuanced understanding of that relationship. Some of the audiences most vulnerable to the health effects of climate change are less likely to say that they do not know whether they are vulnerable: women, African Americans; Hispanics and Latinos; and those with chronic disease or disability. At the same time, people who are higher income and education, with more knowledge of the health effects of climate change in Maryland—qualities typically related to lower social vulnerability—are also less likely to say that they don’t know.

*Climate beliefs.* Our covariates, political ideology and general health risk perceptions, represent heuristics that influence people’s perceptions of vulnerability to the health effects of climate change, and in this case explained half (49%) of the explained variance. But cognitive factors—beliefs—explained another 45%. Climate change belief certainty and information about the potential health effects of climate change in Maryland were two of the largest standardized coefficients, along with general health risk perceptions. Education—considered under social vulnerability factors—also was a significant predictor of vulnerability beliefs, again demonstrating the importance of knowledge and information, though not in a direction that supports the social vulnerability thesis. Knowledge has been shown repeatedly to be a key component of perceptions of climate risk and even behavior [2,51].

*Social vulnerability and health susceptibility.* The demographic and medical condition indicators of social vulnerability and health added 4% of explanatory power to the model. Being African-American was the characteristic of social vulnerability that most strongly influenced climate health risk perceptions. This aligns with previous research demonstrating substantial differences in risk perceptions between whites and non-whites, and especially white males and non-white females [21,22]. Being of Hispanic or Latino ethnicity also was a significant predictor, as was being female, lower income and health-impaired from chronic disease or disability within the household. All of these conditions potentially describe conditions of perceived scarcity—monetary, health, security—that have been hypothesized to fundamentally affect people’s attention to risk and their subsequent decision-making [52].

Being elderly did not affect climate health risk perceptions, although the elderly are at particular risk from extreme heat [53]. Public opinion polls have shown few differences in age on climate change beliefs and risk perceptions; the elderly appear mildly less concerned than middle-aged populations [54]. Previous work has shown that health risk perceptions of older people are elevated for conditions to which they believe they are particularly prone, such as cancer [55]. Climate change-related health impacts may seem to be too far removed in the future for the elderly to find them of personal concern.

*Exposure.* Both of the exposure variables—location in a floodplain or in an urban area (as a proxy for heat island effects)—accounted for only 2% of the variance in the final model. Location in a floodplain was a significant predictor, while urban location was not. While we did not measure experienced flood events or extreme heat, this result resembles the findings by Goebbert and colleagues that people’s perceptions of flooding are more likely to be accurate than their recollection of temperature, which is heavily influenced by the politicization of “global warming” [56]. Other studies have found that people can make accurate judgments about temperature [57]. The proxy nature of our variable—urban location—may have limited our ability to detect this effect.

*Limitations.* The strengths of this study are in the sample’s wide representation of political beliefs and its use of secondary data in combination with survey responses. However, surveys often do not sufficiently reach individuals who are at higher levels of vulnerability [58], and respondents are more likely to be civically engaged than those who do not participate [32]. These conditions reflect the limitations of this study as well. Moreover, the ecology of vulnerability is complex and threat-specific; the measures in this study reflect only broad indicators. The factors for extreme heat vulnerability among the socially vulnerable have been recently explored in detail, calling attention to the importance of social and environmental conditions as well as individual characteristics [53,59].

We speculate that something about the qualities of being vulnerable—due to social, health or location circumstance—makes people more concerned about climate change’s health effects to themselves and their households above and beyond their risk perceptions of flu epidemics, air pollution, chemicals, and polluted water. One possibility is that the amorphous and outsized nature of climate change’s threat—the level to which its effects are both unknown and highly dreaded [60]—makes it distinct from other environmental threats, especially for people who already feel at risk. Further research should explore this hypothesis with these audiences because of the possibility that fear responses may impinge on successful adaptation efforts [61].

Even so, climate change beliefs—including respondents’ ability to identify localized effects of climate change on health—account for more of the variance in the public’s health risk perceptions than do vulnerability factors. This presents a dilemma to communicators as it suggests that while information about the risks of climate change can be valuable, when these messages are targeting vulnerable populations, their heightened sense of risk may cause these messages to backfire. In these groups, messages should perhaps instead focus on specific actions that can be taken to contribute to their sense of control over their health outcomes [61]. The climate change health narratives provided by Ebi and colleagues may be one such approach [62]. If the combination of dread and unknown risk is what makes climate change feel more risky than other environmental threats to these audiences, making the effects more “knowable” and within control may alleviate that fear.

As climate change health impacts, and the need for adaptive public health responses, increase, so too will the need to assist populations of increased vulnerability. Understanding how these groups interpret risks is more important than ever. We note that the effect sizes for indicators of vulnerability are comparatively small compared to those variables that receive more research interest in climate change social science. This suggests that their practical relevance may be limited, but surveys and public health communication often are challenged to reach individuals who are at increased vulnerability [58], so even a small effect may have important policy implications.

## Supplementary Materials

**Table S1:** Regional samples, response rates and margin of error.

Region	Counties	Initial Sample	Refusals	Undeliverable Addresses	Number of Respondents	Response Rate	Margin of Error
Western	Allegany, Frederick, Garrett, Washington	1467	11	97	551	43%	±4.17% points
Central	Baltimore, Carroll, Cecil, Harford, Howard, Montgomery, Baltimore City	2000	14	110	671	38%	±3.78% points
Southern	Anne Arundel, Calvert, Charles, Prince George’s, St. Mary’s	1467	5	90	421	33%	±4.78% points
Eastern	Caroline, Dorchester, Kent, Queen Anne’s, Somerset, Talbot, Wicomico, Worcester	1467	9	180	483	40%	±4.46% points
State	All counties	6401	39	477	2126	38%	±2.1% points

**Table S2:** Descriptive statistics of independent variables (*n* = 2126).

		Min	Max	*M*	*SD*	% Missing Data
Covariates	General risk perception (Factor scores)	−2	1	0	0.92	7.1%
	Political ideology	1	5	3.02	1.12	2.5%
Climate beliefs	Climate change belief certainty	1	9	7.16	1.84	9.5%
	Perceived climate health impacts in Maryland (Scale)	0	4	2.2	1.42	0.0%
Social vulnerability & health susceptibility	Female	0	1	0.62	0.48	0.0%
	African American	0	1	0.22	0.41	2.7%
	Hispanic/Latino	0	1	0.03	0.18	4.8%
	Income	1	9	5.18	2.42	6.6%
	Education	1	5	3.55	1.22	0.0%
	Elderly (Age 65+)	0	1	0.25	0.43	0.0%
	Chronic disease (Scale)	0	2	0.73	0.79	0.0%
Exposure	100-year floodplain	0	1	0.02	0.13	0.0%
	Urban (Heat proxy measure)	0	1	0.86	0.23	0.0%

Weighted to state population distributions. Missing data for covariates, climate beliefs, and income were imputed for the purposes of the final analyses using gender, age and education as matching variables.

**Table S3:** Non-climate health risk perceptions: Factor score variables (*n* = 2126).

*Below Is a List of Potential Risks to People’s Health. How Much of a Risk Do You Feel Each Currently Poses to Your Own Health?*							
	*Frequencies*						
	No risk at all (1)	Minor risk (2)	Moderate risk (3)	Major risk (4)	*n*	*M*	*SD*
Second-hand smoke from tobacco	22.6%	29.3%	21.4%	26.7%	2103	2.52	1.11
Exposure to chemicals, including pesticides, in food and other products	5.9%	23.5%	35.7%	34.9%	2085	3.00	0.91
Air pollution	4.3%	23.4%	39.8%	32.5%	2096	3.00	0.86
Obesity	23.4%	22.1%	20.6%	33.9%	2103	2.65	1.17
Polluted drinking water	16.2%	28.7%	21.1%	34.0%	2074	2.73	1.10
Flu epidemics	5.7%	31.7%	37.8%	24.8%	2097	2.82	0.87

“Don’t know” responses were coded as missing; they represent between 1.1% and 2.4% of responses for each measure and 5.8% total across all 6 measures. “Don’t know” values are left missing and not imputed. Values for other missing data were imputed. They account for between 1.3% to 1.8% of each response for a total of 7.1% across all six measures.
